# Relationship between functional structures and horizontal connections in macaque inferior temporal cortex

**DOI:** 10.1038/s41598-025-87517-3

**Published:** 2025-01-27

**Authors:** Danling Hu, Takayuki Sato, Kathleen S. Rockland, Manabu Tanifuji, Hisashi Tanigawa

**Affiliations:** 1 Department of Neurosurgery of the Second Affiliated Hospital, Interdisciplinary Institute of Neuroscience and Technology, School of Medicine, Zhejiang University, Hangzhou, China; 2 Key Laboratory of Biomedical Engineering of Ministry of Education, College of Biomedical Engineering and Instrument Science, Zhejiang University, Hangzhou, China; 3 MOE Frontier Science Center for Brain Science and Brain-Machine Integration, School of Brain Science and Brain Medicine, Zhejiang University, Hangzhou, China; 4 Laboratory for Integrative Neural Systems, RIKEN Center for Brain Science, Wako, Saitama Japan; 5 Communication Future Design Center, Fukushima University, Fukushima, Fukushima Japan; 6 Department of Anatomy and Neurobiology, Chobanian & Avedisian School of Medicine, Boston University, Boston, MA USA; 7 Department of Life Science and Medical Bio-Science, Faculty of Science and Engineering, Waseda University, Shinjuku, Tokyo Japan

**Keywords:** Intrinsic signal optical imaging, Anterograde tracer, Monkey, Non-human primate, Intrinsic connections, Extrastriate cortex, Object vision

## Abstract

Horizontal connections in anterior inferior temporal cortex (ITC) are thought to play an important role in object recognition by integrating information across spatially separated functional columns, but their functional organization remains unclear. Using a combination of optical imaging, electrophysiological recording, and anatomical tracing, we investigated the relationship between stimulus-response maps and patterns of horizontal axon terminals in the macaque ITC. In contrast to the “like-to-like” connectivity observed in the early visual cortex, we found that horizontal axons in ITC do not preferentially connect sites with similar object selectivity. While some axon terminal patches shared responsiveness to specific visual features with the injection site, many connected to regions with different selectivity. Our results suggest that horizontal connections in anterior ITC exhibit diverse functional connectivity, potentially supporting flexible integration of visual information for advanced object recognition processes.

**Supplementary Information:**

The online version contains supplementary material available at 10.1038/s41598-025-87517-3.

## Introduction

Horizontally projecting axon collaterals (also known as “horizontal axons”) are ubiquitously observed in various regions of the cerebral cortex. They extend parallel to the pial surface for several millimeters or more and branch at their terminals to form dense clusters of axon terminals called patches^1–6^. These neuronal connections, known as horizontal or intrinsic connections, are thought to be closely related to cortical functional structure. Specifically, in macaque primary visual cortex (V1), horizontal axons form patches averaging 200–300 μm in diameter^6,7^, which corresponds to the width of a single orientation column. The distance between these patches, ranging from 0.5 to 1 mm^6,8,9^, approximates the span of a full cycle of orientation columns^10^. Studies correlating patch distribution with functional and anatomical maps have reported that horizontal axons tend to predominantly connect sites that share stimulus selectivity (such as orientation preference or ocular dominance)^7,11,12^ or similar cytochrome oxidase (CO) activity, which is associated with specific visual features^13–18^. This specificity, often referred to as the “like-to-like” connectivity rule, is thought to be facilitated by a Hebbian “fire together, wire together” mechanism^19–21^. Horizontal connections in V1 are known to extend beyond the cortical point spread, which is the cortical region activated by minimal visual stimuli, suggesting they provide an anatomical framework for contextual interactions beyond the classical receptive field, such as contour integration^22^.

Recent studies have suggested that this like-to-like rule for horizontal connections may not fully capture the complexity of their connectivity. Even early studies, while recognizing a tendency for like-to-like bias in horizontal connections, simultaneously showed that a substantial proportion of these connections (20–30% or more) link sites with different orientation preferences, ocular dominances, and CO activities^7,12,18^. A similar result has been reported for the relationship between synaptic bouton distributions and orientation maps^23^. It has also been shown that the degree of iso-orientation bias varies significantly between individual cells^24^, and decreases with increasing projection distance^25^. Studies using local visual stimuli^26^ and focal optogenetic activation^27^ have shown that the horizontal propagation of activation is not specific to orientation. In cortical areas beyond V1, such as V2 in squirrel and macaque monkeys and area MT in owl monkeys, while there is a tendency for sites with similar orientation preferences or the same type of CO compartments to link, this tendency is weaker than in V1 and varies from site to site^28–30^. These findings suggest that the tendency for “like-to-like” connections represents one organizing principle, but it is not an absolute rule, and other functional characteristics may also play a role in the formation of these connections. Thus, the functional specificity of horizontal connections is characterized by diversity rather than uniformity^31–33^.

Moving to higher cortical regions, cytoarchitectonic area TE^34^, located in the anterior part of the macaque inferior temporal cortex (ITC), is situated at the final or near-final stage of the ventral visual pathway, which is a highly interconnected complex cortical visual processing system for object recognition^35^. Compared to V1, neurons in TE have much larger receptive fields (> 15–40° in diameter) and respond selectively to more complex visual stimuli, such as faces, body parts, and combinations of visual features^36–38^. Neurons with similar response properties cluster together to form functional columns^39,40^, and a single visual stimulus can activate multiple columns^41,42^. Horizontal axons in the TE cortex, as in V1, form terminal patches with widths similar to those of functional columns^43^. However, their projection distances are much longer than those in V1 (up to 8 mm or more), and the sizes and intervals of the patches are more irregular^6^. It has been proposed that visual objects are represented by combinations of multiple columns in TE^41,44^, and horizontal axons are thought to contribute significantly to interactions between these columns. However, it is still unclear which functional columns horizontal axons connect in the TE. Furthermore, while fMRI studies have revealed functional domains such as multi-millimeter ‘face patches’ in the ITC^45^, our understanding of the intrinsic architecture within these domains is still limited.

In this study, we investigated the functional specificity of horizontal connections in TE by examining the relationship between stimulus response maps and patterns of horizontal axon terminal patches. We mapped object stimulus responsiveness in the TE region using intrinsic signal optical imaging (ISOI) and multi-unit activity recordings, then injected an anterograde tracer into an activated site. Analysis of the relationship between functional maps and labeled axon patch patterns revealed that horizontal axons in TE do not preferentially connect sites with similar stimulus selectivity. This suggests that horizontal connections in higher-order cortical areas may deviate from a simple like-to-like rule, potentially encompassing more complex and diverse connectivity.

## Results

### Mapping object stimulus responses in TE using intrinsic signal optical imaging

**Fig. 1:**
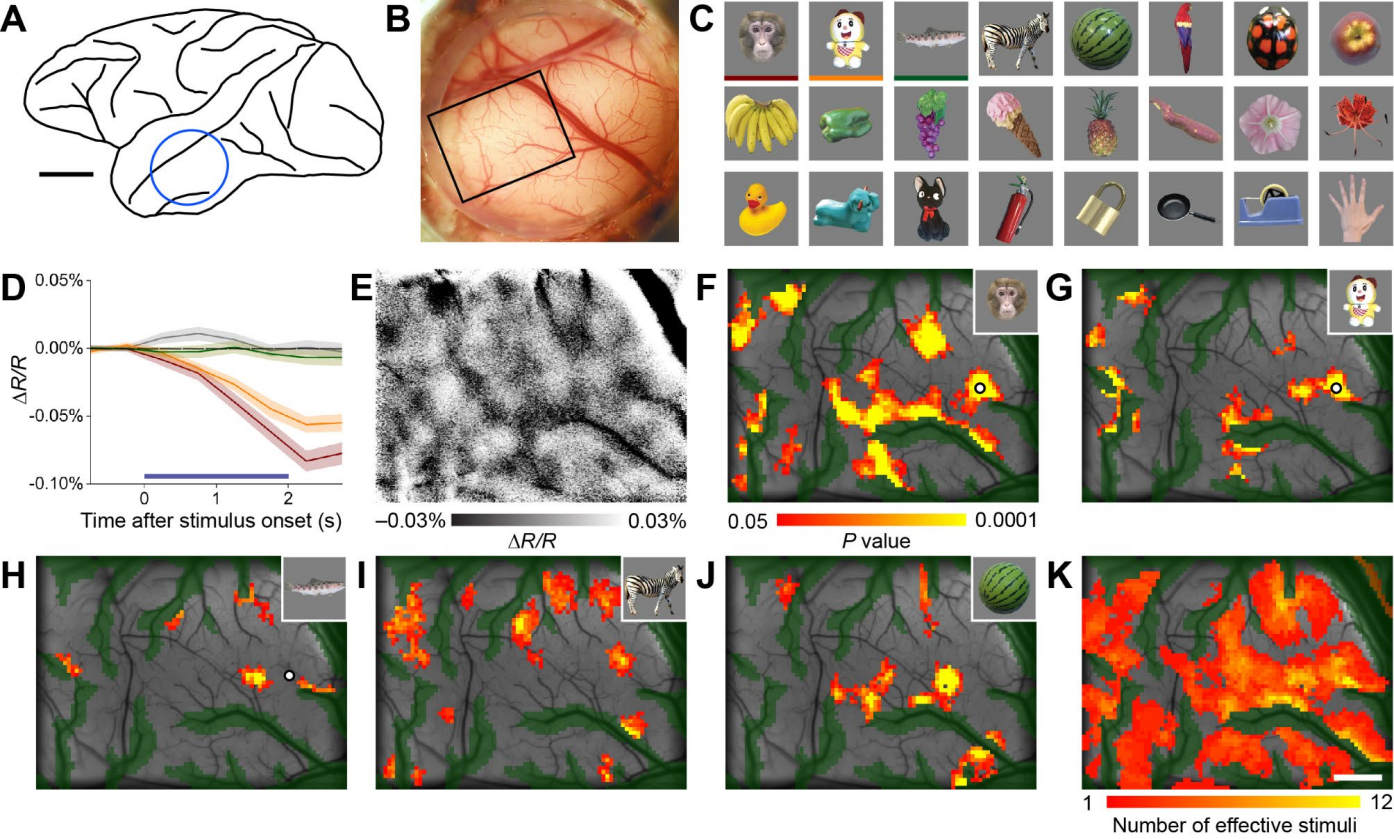
Mapping object stimulus responsiveness in the macaque inferotemporal cortex using intrinsic signal optical imaging. (**A**) Schematic representation of the lateral view of the postmortem brain of monkey M4. The blue circle indicates the position of the recording chamber implantation. Scale bar, 1 cm. (**B**) View of the cortical surface including TE through the recording chamber. The black rectangle indicates the imaging area. (**C**) Example of a set of object stimuli used in the ISOI and MUA recordings. (**D**) Time course of the average change in light reflectance (Δ*R/R*) under 630-nm illumination induced by different object stimuli at a specific location within the imaging area. The color of the lines corresponds to the color of the lower bars of the visual stimuli in (**C**). The gray line indicates changes in the blank condition. The shaded areas represent the SEM across presentations (*n* = 32). An increase in neural activity leads to an increase in light absorbance, resulting in a decrease in reflectance. (**E**) A single condition map subtracting changes in the blank condition from changes in response to the monkey face stimulus on a per-pixel basis. Darker areas indicate a response to the stimulus. Note that in large blood vessels and their surroundings, image saturation may occur due to exceptionally large signal changes (e.g., upper right). (**F**–**J**) Response maps showing the distribution of neural activity on the cortical surface in response to different object stimuli. The stimuli used are shown in the upper left corner. Colored areas indicate significant excitatory reflectance changes compared to the blank condition (two-tailed Wilcoxon rank-sum test, see “Methods” for details on multiple comparison corrections). Green shaded areas indicate pixels with large trial-to-trial variability near large vessels (see “Methods”). Black crosses in (**F**–**H**) indicate sample locations (120 μm in diameter) for the time courses in panel D. (**K**) A map showing the number of stimuli from the set in C (including 24 stimuli) that elicited significant responses. Scale bar, 1 mm on (**E**–**K**).

We used intrinsic signal optical imaging to generate maps of responsiveness to a set of object images in the dorsal part of area TE (Fig. 1A and B) in anesthetized macaque monkeys. The set of visual stimulus images consisted of different categories, such as animals, fruits, plants, stuffed animals, and gadgets, with a range of colors and shapes (Fig. 1C). Many of the objects were ones that the monkeys had not encountered while awake. Within the imaged area, responses to the object stimuli varied by location. At some locations, specific stimuli from the set elicited strong active optical responses (decreases in reflectance), while other stimuli elicited no response at all (Fig. 1D). Single-condition maps were constructed by subtracting the response to the blank condition from the optical response to each individual stimulus on a pixel-by-pixel basis (single-condition maps, Fig. 1E). In these maps, areas responding to stimuli appeared as dark spots. Meanwhile, large positive or negative signal changes were observed near large blood vessels. Statistical analysis identified the distribution of spot-like activations (active spots) elicited by the stimuli (response maps; Fig. 1F-J; see Methods for multiple comparison correction in statistical maps). The distribution of active spots in the response maps showed substantial agreement across imaging sessions performed on different days (Figure S1). In most cases, individual stimuli in the set elicited different patterns of activation, indicating that many locations within the region responded to some extent to one or more of the visual stimuli (Fig. 1K and S2). This suggests that the selected set of stimuli was sufficiently diverse to activate these areas ubiquitously.

### Functional connectivity of horizontal axons with terminal patches revealed by imaging

**Fig. 2:**
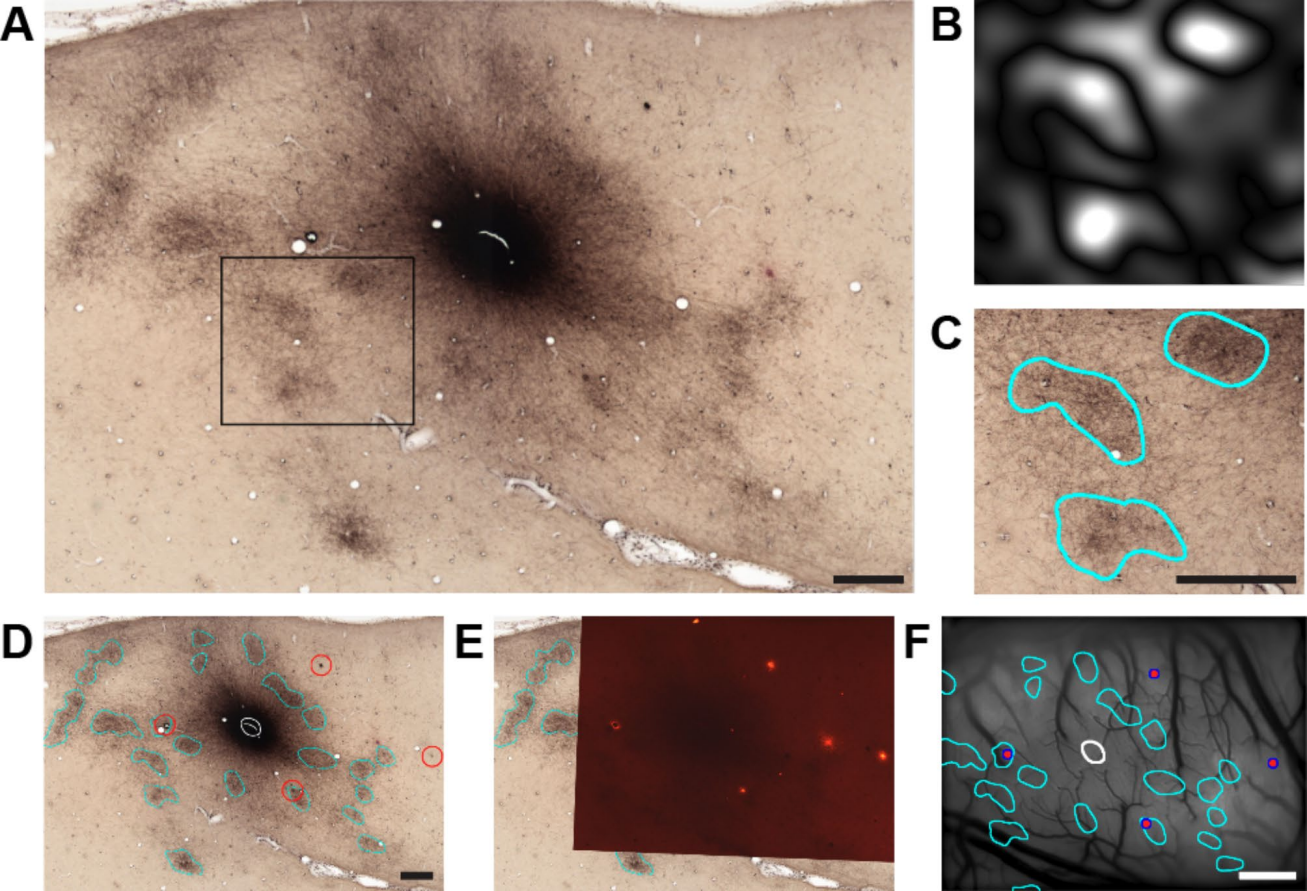
Identification of terminal patches of the horizontal axons and their alignment with functional maps. (**A**) A tangential section at approximately 950-µm depth from the cortical surface, cut through mainly the supragranular layer, demonstrating horizontal axon projections labeled with an anterograde tracer. The dark central area includes the tracer injection site and its dark halo. (**B**) An image within the black frame in A, processed using the Difference-of-Gaussian algorithm. The outlines of the terminal patches are highlighted as dark troughs, corresponding to the maximum local brightness change. (**C**) Outlines of terminal patches were drawn from the processed image in (**B**) (cyan outlines). (**D**) Terminal patches were delineated around the injection site in the tangential section (cyan outlines). In addition, the extent of the injection site was estimated from the spread of the distribution of descending axon bundles labeled in deeper sections (white outline). Traces of the metal needle are indicated by red circles. (**E**) Fluorescence microscopy image of the same section as in (**D**), overlaid onto the image in (**D**), with their positions aligned. DiI-labeled spots made by the electrode or metal needle are visible. (**F**) By linearly transforming the section image to align the DiI-labeled spots on the section with the positions of metal needle insertions on the cortical surface (magenta dots), outlines of terminal patches (cyan outlines) and the injection site (white outline) are projected onto the functional map, integrating anatomical and functional data. For a detailed description of the procedure shown in this figure, see Methods. Scale bar: 0.5 mm on (**A**, **C**, **D**), and 1 mm on (**F**).

To examine the distribution of terminal patches of horizontal axons projecting from an activation spot revealed by imaging, we injected an anterograde tracer into one of the spots. The injection was aimed at the depth of the supragranular layers, where horizontal connections are more clustered^46^ and are thought to reflect the signals in ISOI more. After a survival period following injection, the animal was perfused and tangential sections of the TE cortex containing the injection site and the imaged region were prepared. The sections were histologically stained for tracer-labeled horizontal axons. In the sections, patchy clusters of labeled axon terminals (terminal patches) were observed around the injection site (Fig. 2A). An edge detection algorithm based on luminance changes in the image was used to delineate the outlines of the terminal patches (Fig. 2B–D). The traces of DiI-coated needles inserted perpendicular to the cortical surface before perfusion were observed in the sections under bright-field (Fig. 2D, red circles) and fluorescence microscopy (Fig. 2E). The image containing the injection site and the terminal patch pattern was linearly transformed to align the positions of the needle traces on the sections with their insertion positions on the imaging map (Fig. 2F, magenta dots) and superimposed on the imaging region (Fig. 2F, see Methods for details of the series of operations).

**Fig. 3:**
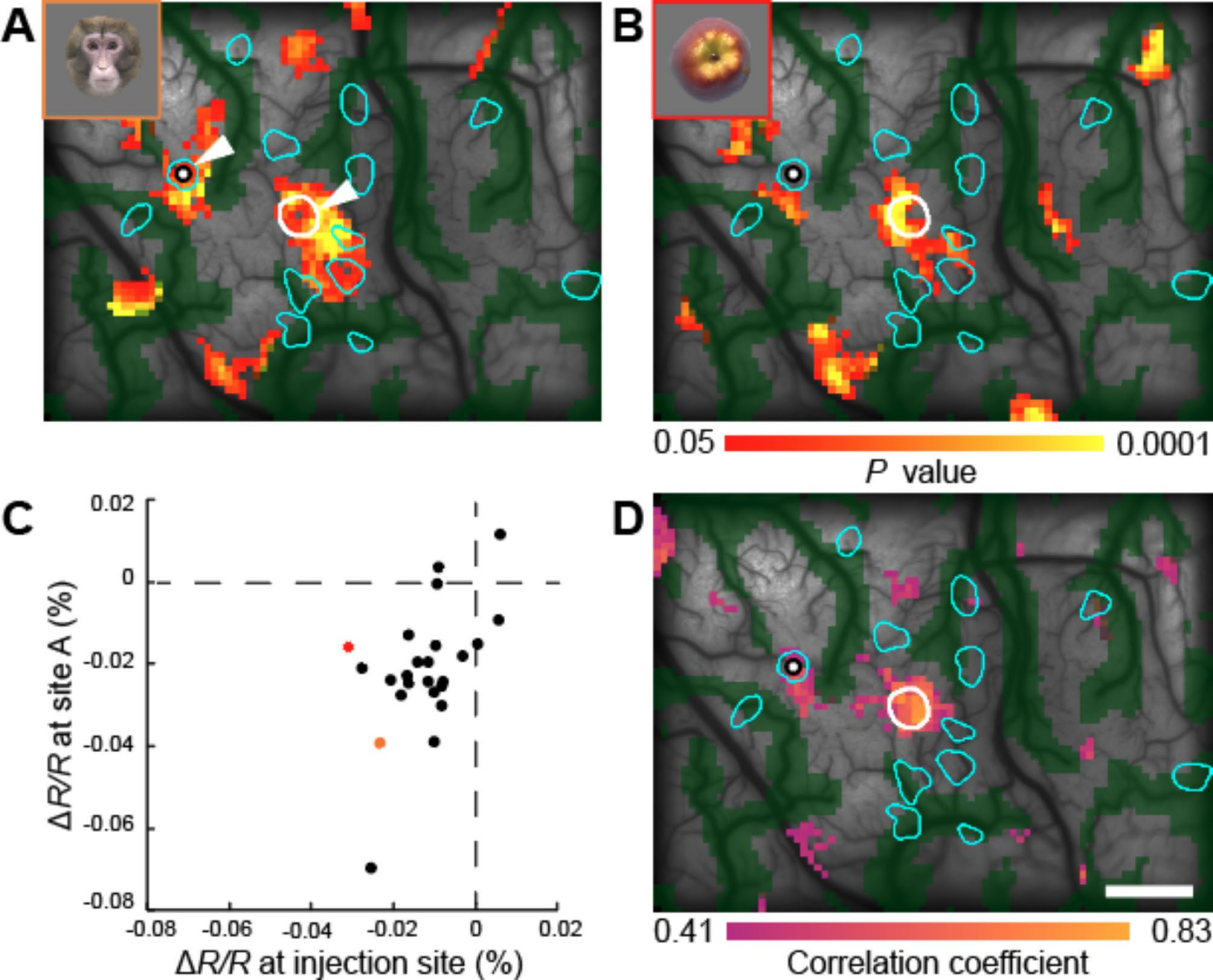
Evaluation of object stimulus response specificity of horizontal axonal patches. (**A**, **B**) Response maps to object stimuli (shown in the upper left inset) in monkey M4, overlaid with the injection site (white outline) and patch regions (cyan outlines). (**C**) Scatter plot of the average response of the injection site and the mean response of an arbitrary site (**A**) (indicated by white dots in (**A**) and (**B**)) to the object stimuli within the stimulus set. Responses to the stimuli used in (**A**) and (**B**) are indicated by yellow and red dots, respectively. The responses to the stimuli at both sites were significantly correlated (Spearman’s rank correlation, *ρ* = 0.49, *P* = 0.016). (**D**) In the response correlation map, pixels with responses significantly correlated with the mean response of the injection site to the object stimuli within the stimulus set are color-coded according to the color scale (Spearman’s rank correlation, *P* < 0.05). Scale bar: 1 mm. Correlation maps for other monkeys are shown in Fig. S3. Scale bar: 1 mm.

If horizontal axons with terminal patches preferentially connect sites with similar stimulus selectivity, object stimuli that activate the injection site should also tend to activate the patch regions. Indeed, in the response maps to object stimuli, we found examples of patches where both the injection site and parts of the patch regions were simultaneously activated by the same object stimuli (Fig. 3A, arrowheads). However, many patches did not share stimulus responsiveness with the injection site (Fig. 3A, B). We then examined whether the same object stimuli that significantly activated the injection site also activated individual patch regions. As a result, out of 43 patches identified within the imaging area in four monkeys, 17 patches were activated by at least one of the object stimuli in the same way as the injection site (two-tailed Wilcoxon rank-sum test, *P* < 0.05, Bonferroni corrected for the number of stimuli that significantly activated the injection site). However, this was not significantly greater than the number expected by chance when randomly shifting the patch locations (one-tailed randomization test, 10,000 iterations, *P* > 0.187). Next, we examined whether the response profiles to the stimulus set in individual patch regions correlated with those at the injection site. Of the 43 patches identified, only five had response profiles that correlated with those at the injection site (Spearman’s rank correlation, *P* < 0.05, uncorrected). We also visualized regions within the imaged area where the response profiles to the stimulus set were similar to those at the injection site (response correlation map). In the response correlation map, pixels with responses to the stimulus set that were significantly correlated with the responses at the injection site were color-coded (Fig. 3C, D; Spearman’s rank correlation, *P* < 0.05). In four injection cases from 4 monkeys, an average of 10.11 ± 4.77% (mean ± SD, *n* = 4) of the patch regions within the imaging area overlapped with regions where the response correlated with the injection site (Fig. 3D and S3). If horizontal axons preferentially connect sites with similar stimulus selectivity, patches should overlap more with regions where responses correlate with the injection site. However, in none of the four cases was this overlap ratio significantly greater than the overlap ratio observed by chance (one-tailed randomization test, 10,000 iterations, *P* > 0.05). These results suggest that while horizontal axons with terminal patches can connect regions responding to specific object stimuli, many connect regions with different selectivities, and there is no tendency to preferentially connect regions with similar object selectivity.

**Fig. 4:**
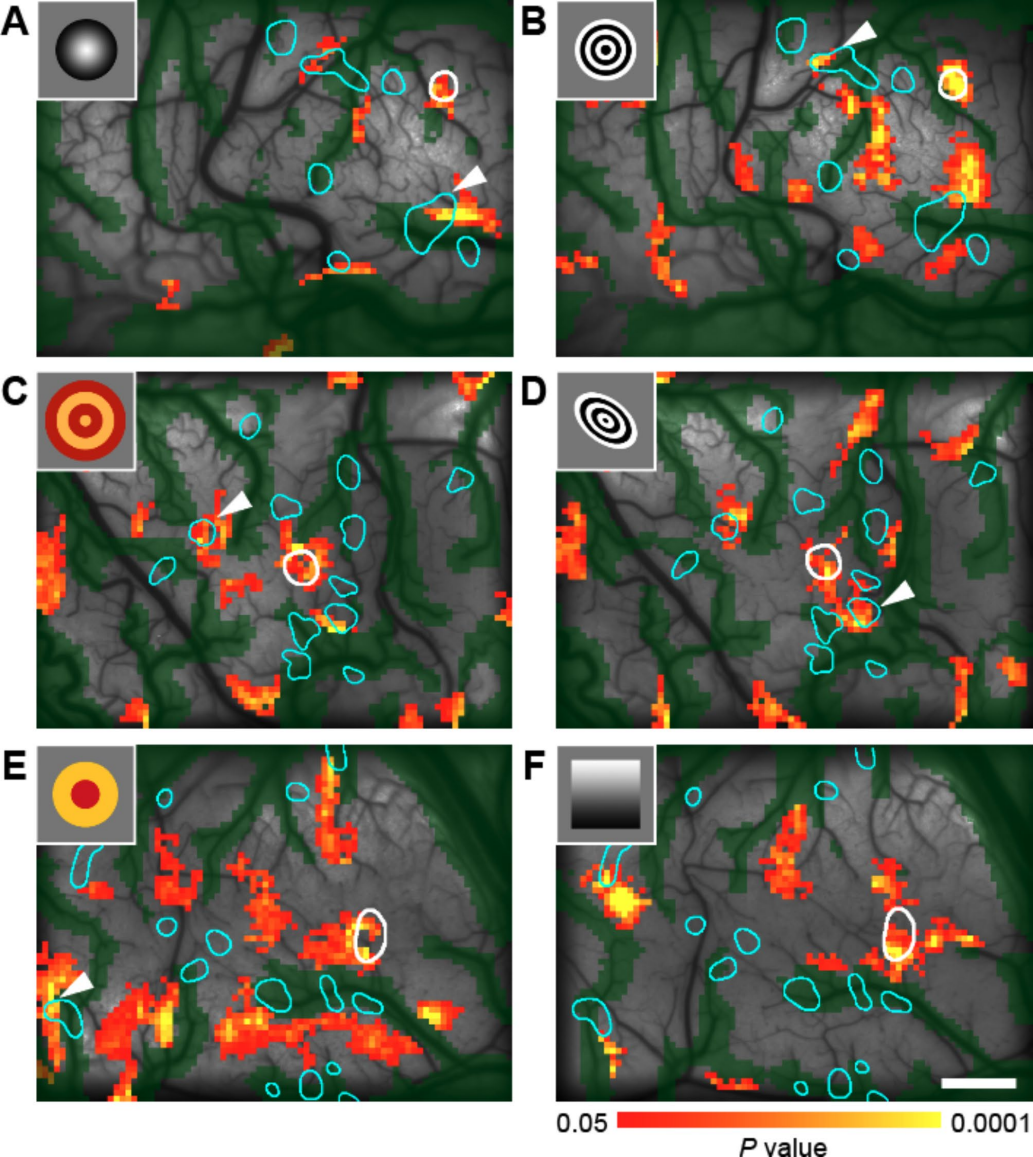
Response specificity of horizontal axonal patches to simplified visual stimuli. (**A**–**F**) Response maps to simplified visual stimuli in monkeys M1 (**A**, **B**), M4 (**C**, **D**), and M3 (**E**, **F**), overlaid with the injection site (white outline) and patch regions (cyan outlines). Here, examples of stimuli that significantly activated the average signal of the injection site are selected (two-tailed Wilcoxon rank-sum test, *P* < 0.05). Arrowheads indicate patches whose average signals were significantly activated by the stimuli (two-tailed Wilcoxon rank-sum test, *P* < 0.05, Bonferroni corrected for the number of stimuli). Scale bar: 1 mm.

In a previous study using ISOI in TE, spots were found that were activated not only by the presence of specific features in objects but also by the absence of specific features in objects^41^. This complicates the interpretation of spots activated by objects containing multiple features. Therefore, we overlaid terminal patches on activation maps obtained by ISOI using visual stimuli composed of simpler features (Fig. 4). We prepared a set of visual stimuli by referring to examples of simplified visual stimuli from previous studies^42^ that were sufficient to maximally activate individual neurons identified in unit recordings in TE (see image examples in the upper left of each panel in Fig. 4). Some of these simplified stimuli were confirmed to activate spots revealed by ISOI (Fig. 4). We then examined whether, in cases where the injection site was significantly activated by a simplified stimulus, the stimulus also activated individual patch regions. As a result, of 43 patches identified in four monkeys, 14 patches were activated by simplified stimuli in the same way as the injection site (two-tailed Wilcoxon rank-sum test, *P* < 0.05, Bonferroni corrected for the number of stimuli that significantly activated the injection site). This was also not significantly greater than the number expected by chance when patch positions were randomly shifted (one-sided permutation test, 10,000 iterations, *P* > 0.4693). This suggests that while a minority of horizontal axons with terminal patches connect regions that respond to specific simplified visual features, they do not show a tendency to preferentially connect such regions.

This suggests that a certain proportion of horizontal axons with terminal patches connect regions that respond not only to certain object stimuli, but also to certain simplified visual features.

### Electrophysiological validation of functional maps and horizontal axonal connectivity

**Fig. 5:**
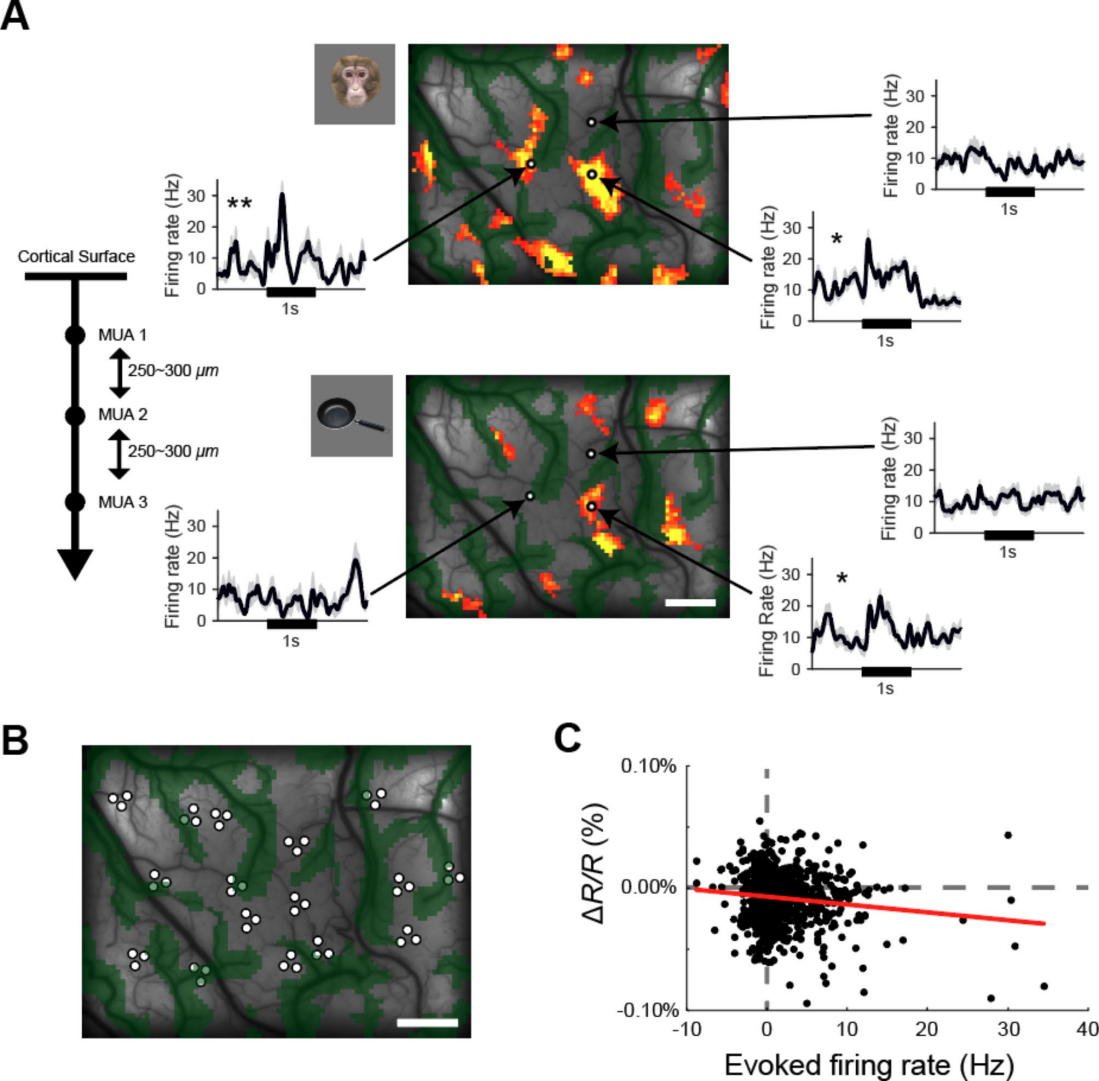
Correlation between optical imaging and electrophysiological responses to object stimuli. (**A**) Left: Multi-unit activities (MUAs) were recorded at three depths, each 250–300 µm apart, starting from the depth at which spike activity was first detected using a microelectrode inserted vertically into the cortical surface. These MUAs were then averaged to obtain the mean MUA for that insertion site. Right: Peristimulus time histograms (PSTHs) of the average MUAs evoked by two different object stimuli (top: monkey face; bottom: frying pan) recorded in monkey M3 (*n* = 12 trials, error bars represent SEM). The three recording sites are shown as white dots on the response maps for each stimulus. The stimulus presentation period (1 s) is indicated by a black horizontal bar in the PSTHs. Asterisks indicate the significance of the stimulus-evoked response compared to the pre-onset period (Wilcoxon signed-rank test, *P < 0.05, **P < 0.01). (**B**) All electrode insertion sites (white dots) within the imaging region in monkey M3. (**C**) Correlation between the mean response of the average MUA in electrophysiological recordings and the mean change in optical reflectance (Δ*R/R*) in imaging at the electrode insertion positions, in response to the object stimuli within the stimulus set. The reflectance change for each stimulus at each insertion position was plotted against the evoked MUA response at the same position.” Recordings in regions with large reflectance variations near large blood vessels were excluded from this plot. The regression line (red line) was *y* = − 6 × 10^− 6^*x* − 7 × 10^–5^. Scale bar: 1 mm. Results for monkey M4 are shown in Fig. S4.

To electrophysiologically verify the relationship between the functional maps revealed by imaging and the terminal patches, we recorded multi-unit activity (MUA) responses to object stimuli within the imaged area in two monkeys (M3 and M4) after imaging but before tracer injection. Microelectrodes were inserted vertically into the cortical surface at sites inside and outside the active spots. Representative MUAs recorded from sites that showed significant optical reflectance changes to a stimulus also showed significant activation to the same stimulus (Fig. 5A). In contrast, these MUAs did not show significant responses to a stimulus that did not elicit significant reflectance changes in imaging. The visually evoked MUAs recorded at sites within the imaging area of each of the two monkeys in response to the object stimuli in the set showed a weak but significant negative correlation with the reflectance changes at these sites in imaging (Fig. 5B, C and S4; Spearman’s rank correlation, M3: ρ = − 0.073, *P* = 0.035; M4: ρ = − 0.198, *P* = 9.69 × 10^− 10^). This indicates that the functional maps obtained by imaging for object stimuli reflect, to some extent, the spiking activity of neurons.

**Fig. 6:**
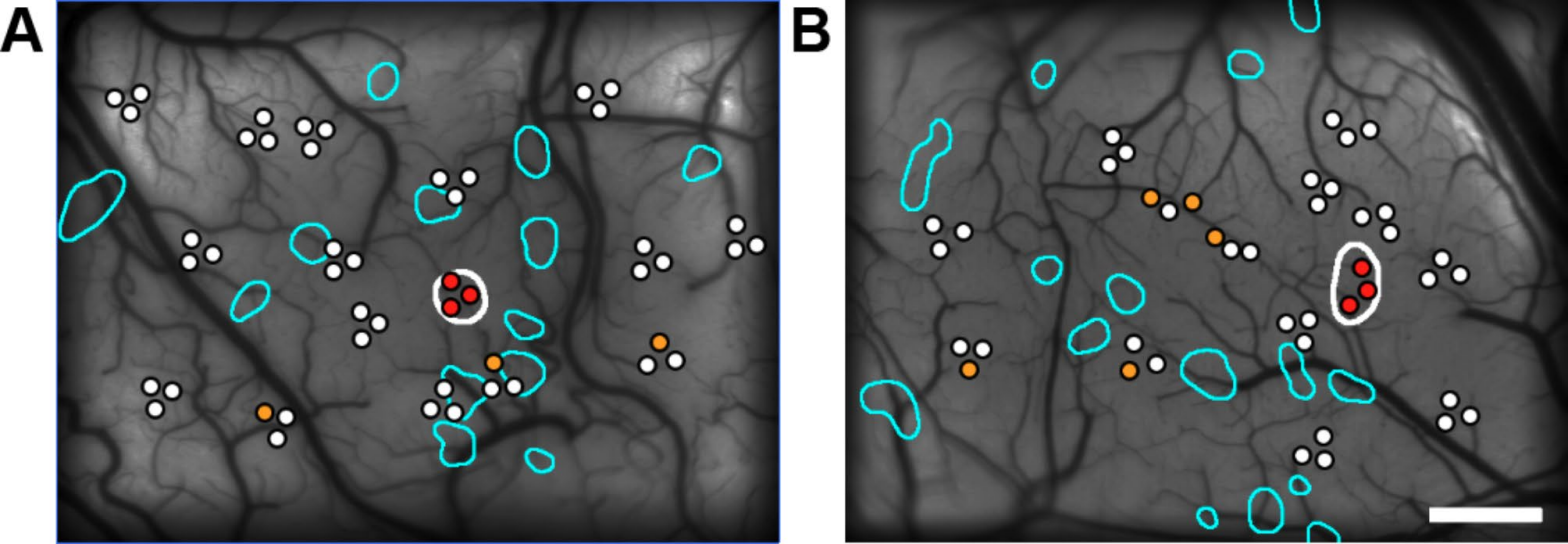
Comparison of object stimulus selectivity between electrophysiological recording sites and the injection site. (**A**, **B**) Among the electrophysiological recording sites (white dots) within the imaging area in monkeys M3 (**A**) and M4 (**B**), those contained within the injection site (red dots) and those with mean MUA responses to the object stimuli within the stimulus set that significantly correlated with the mean MUA response of the injection site (orange dots) are color-coded (Spearman’s rank correlation, *P* < 0.05). The injection site (white outline) and patch regions (cyan outlines) are superimposed. Scale bar: 1 mm.

Next, we identified the locations on the imaging map where multi-unit activity (MUA) responses to object stimuli in the set were correlated with those at the injection site (Fig. 6). In two monkeys, multiple sites were observed where MUA responses were significantly correlated with those at the injection site (Spearman’s rank correlation, *P* < 0.05), but these sites were not located within the terminal patch regions. Although none of the electrode insertion sites were located directly within the patch regions, precluding direct confirmation of the object selectivity of these regions, this result supports the imaging findings, suggesting that horizontal axons with terminal patches do not tend to preferentially connect sites with similar object selectivity.

## Discussion

Using a combination of intrinsic signal optical imaging, electrophysiological recordings, and anatomical tracing, we show that terminal patches of horizontal axons in TE, located in the anterior part of the macaque inferior temporal cortex (ITC), exhibit diverse connectivity patterns. While some horizontal axons in TE connect regions that respond similarly to certain visual features, many others connect regions with different object selectivity. This finding contrasts with the “like-to-like” connectivity rule reported in V1. Our data suggest that the functional organization of horizontal connections in higher cortical areas such as TE may follow different principles from those in early visual cortex. Such connectivity of horizontal axons may indicate a more flexible and integrative intrinsic network capable of supporting the diverse, context-dependent processing required for advanced visual functions such as object recognition and categorization.

Based on the finding that the tendency for “like-to-like” connections is weaker in higher visual areas such as V2 and MT compared to V1^29–31^, it was predicted that the connection specificity reported in V1 would not be similarly observed in TE. Nevertheless, previous anatomical studies, including our own, have repeatedly suggested that horizontal axons in TE might connect columns with similar stimulus selectivity, because the size and spacing of horizontal axon terminals roughly matched those of columns of similar stimulus selectivity, and most of the cells projecting horizontal axons were excitatory pyramidal cells^6,43,46^. Below, we review the results of previous studies and consider the reasons why “like-to-like” connectivity was not observed in our study.

An electrophysiological study reporting the functional column structure of TE^40^ used a stimulus set that included visual stimuli with combinations of specific features (such as shape, color, and texture) designed to activate neurons. This study revealed multiple distant neuron clusters with similar stimulus selectivity, as observed along the tangential electrode penetration in TE. While the responsiveness to these stimulus sets was likely similar among those clusters, responsiveness to other visual stimuli was not examined. In two subsequent studies using predetermined stimulus sets that included various object stimuli, the average correlation coefficients of stimulus selectivity from isolated unit activities of adjacent neuron pairs thought to be within the same cortical column were 0.08^47^ and 0.11–0.15^40^, respectively. These coefficients were significantly higher than those for distant neuron pairs thought to be in different columns, but were still weak. No significant correlations were observed for distant neuron pairs separated by more than 600 μm^39^. These results suggest that while neurons within a single column or specific distant columns may share responsiveness to certain specific visual features, their responsiveness to other visual stimuli is likely to differ greatly.

In the present study, both optical imaging and electrophysiological recordings were used to identify sites with stimulus selectivity similar to the injection site. Optical imaging reflects hemodynamics associated with local neuronal population activity, and our physiological recordings used the average of MUAs recorded from the same vertically inserted electrode for analysis. Therefore, these correlation analysis results likely reflect the similarity of average activities of local neural populations in response to the prepared stimulus set. This approach failed to demonstrate any tendency for horizontal axons to connect sites with such similarity. Regarding whether horizontal axons tend to connect sites that share responsiveness to specific visual features, we found that while nearly 30% of patches indeed shared responsiveness to some visual features, others did not. The latter patches may have shared responsiveness to other visual features that were not tested. Our conclusion is that not all horizontal connections from a site connect to sites sharing a specific stimulus feature. Rather, the shared stimulus features differ for each projected patch, likely reflecting the diverse stimulus selectivity of neurons within columns.

In the TE, terminal patches of horizontal axons are formed by postnatal day 7, and the overall patch pattern does not change significantly until adulthood^48^. In macaque V1 and V2, horizontal axon projections become patchy as early as 3 weeks before birth^49^. This suggests that patch formation of horizontal axons is likely established in utero across visual areas. What is the mechanism by which horizontal axon patches are formed prenatally? In mice, retinal waves, patterns of spontaneous neural activity in the retina before eye opening, have been shown to propagate throughout the visual system^50^, and computational modeling in cats and monkeys has proposed that the clustered horizontal projections in V1 are formed by structured activity from retinal waves^51^. Although it is unknown whether retinal waves prenatally influence the development of higher visual areas such as TE, they may propagate through the visual hierarchy and have an effect on horizontal projections. The presence of retinotopic organization in the ITC by postnatal day 10^52^ suggests that patterned activity, possibly derived from retinal waves, influences ITC’s prenatal organization. Based on this, it has been proposed that the higher visual cortex, including TE, initially has a proto-architecture for retinotopy and receptive field size at birth that provides a scaffold for the development of category-selective regions such as face patches^53^. This proto-architecture may extend to the column-level functional organization, possibly guiding prenatal formation of horizontal axon patches through Hebbian-like mechanisms.

An fMRI study has revealed the functional organization for low-level visual features in ITC at 1 month postnatally, with face-selective patches (functional domains) forming at about 200 days postnatally^54^. The findings that face perception experience is necessary for face-patch formation^55^ and intensive training with novel shapes creates new selective regions^56^ demonstrate that visual experience shapes the development of category-selective regions in ITC. These changes in domain-level organization likely involve concurrent modifications in column-level functional organization. If horizontal axon patches are formed prenatally based on proto-architecture and their pattern does not change postnatally, while column-level organization undergoes substantial reconstruction based on visual experience, they are thought to no longer exhibit strict “like-to-like” connectivity.

It has been suggested that the stimulus selectivity of each neuron in TE columns is characterized by two types of response properties: those common to all cells within the column and those that are cell-specific^39^. In V1, thalamic inputs are thought to establish basic orientation preferences, while horizontal connections serve to refine orientation selectivity, provide contextual modulation, and integrate information^57^. Applying this idea to TE, the common response properties of neurons within a column may be formed by inputs from lower visual areas and the pulvinar thalamus^35,58^, while horizontal connections likely contribute to the formation of cell-specific, diverse response properties needed to refine these common properties. After birth, while the overall pattern of horizontal axon patches in TE remains stable, significant refinement occurs through increased bouton density and axon branching within patches^48^. As observed in adult macaque V1 where small branches of horizontal axons continuously remodel^59^, this refinement in TE likely reflects experience-dependent plasticity. Throughout this developmental process, horizontal connections are thought to contribute to the formation of more cell-specific and diverse response properties while maintaining a foundation of common response characteristics. Such response properties within a column would allow for a broader, more flexible, and noise-robust object representation^39^. The continuous refinement of horizontal connections within patches may function as a mechanism to adapt to environmental changes.

Based on findings that facilitatory interactions between V1 neurons with similar orientation selectivity are enhanced during contour detection tasks^60^, and that V1 neurons exhibit task-dependent geometric selectivity^61^, it has been proposed that horizontal connections are subject to top-down control. Functionally, this might involve attention and task-specific demands, enabling dynamic connectivity changes to gate inputs under different behavioral contexts^22^. Extending this concept to TE, which presumably receives stronger top-down influences from the prefrontal cortex and medial temporal lobe structures such as the perirhinal cortex and hippocampus^35^, we propose that horizontal connections between columns representing different features may play a crucial role in the behavior-dependent modulation of visual information processing. These connections could dynamically adjust their efficacy in response to top-down signals related to attention, memory, and category representations, thereby facilitating flexible and context-dependent object recognition^62–65^. This dynamic modulation of horizontal connections in TE may contribute to the remarkable adaptability of the primate visual system in recognizing objects across various contexts and task demands^66^.

Another potential role of horizontal connections in TE involves the modulation of local inhibitory connections. Inhibitory interneurons in TE significantly contribute to the formation of stimulus selectivity^67^. While the projections of these inhibitory neurons in TE are largely confined to within approximately 1 mm^46^, long-range horizontal axons can modulate neuronal activity in distant columns by influencing the activity of these inhibitory neurons^68^. Studies have suggested that activation of certain visual feature representations in TE can lead to suppression of other representations^41,69^, and such suppressive effects may be, at least in part, mediated by inhibitory interactions involving horizontal connections. Therefore, the role of horizontal connections in TE likely involves not only excitatory interactions but also modulation of the local inhibitory network, both of which may play crucial roles in integrating different feature representations and executing complex computations underlying object recognition.

While our study provides important insights into the functional organization of horizontal connections in TE, it is important to acknowledge several limitations of our approach. The stimuli included in the stimulus set used in this study were selected from various categories containing different shapes and colors, but their number was limited to 18–24. This may not fully represent the variety of object categories that TE can potentially encode and may lead to results biased by the specific stimulus set used. In our previous electrophysiological studies using predetermined stimulus sets containing object stimuli, approximately 100 stimuli were used^39^. In this study, we were unable to present such a large variety of stimuli due to time constraints, as optical imaging requires a 10-s interstimulus interval to allow the signal to return to baseline. Nevertheless, given that the focus of this study was on whether horizontal axons show a tendency to connect sites with similar stimulus selectivity, no such tendency was observed even with our limited stimulus set. Therefore, we believe that the conclusion would not have changed significantly even if more stimuli had been used.

It should also be noted that our experiments were performed under anesthesia, which may affect neural responses. In addition, the visual responses in TE were recorded under conditions where single stimuli were presented on a gray background, which is different from natural environments that typically involve complex and dynamic visual scenes. Future studies using awake behaving animals and more naturalistic stimuli may provide further insight into the functional organization of horizontal connections in the TE under ecologically valid conditions.

## Methods

### Animals

Four hemispheres of four macaque monkeys (*Macaca mulatta*; weight 4.6–8.1 kg; two females and two males) were used in the study. The monkeys were purchased from HAMRI Co., Ltd., Japan and housed at the RIKEN Center for Brain Science. We performed ISOI and electrophysiological recording experiments on two monkeys and ISOI only on the other two monkeys. All experiments were performed under anesthesia. This study was conducted in accordance with the ARRIVE guidelines for reporting animal research. The experimental protocol was approved by the RIKEN Experimental Animal Committee, and all animal procedures were in accordance with the Act on Welfare and Management of Animals in Japan and the National Institutes of Health Guide for the Care and Use of Laboratory Animals.

### Anesthesia

In all experiments, monkeys were initially anesthetized with an intramuscular injection of ketamine (5.8 mg/kg). During the initial surgical procedure to implant a solid head post and recording chamber, the monkeys were deeply anesthetized with an intraperitoneal injection of pentobarbital sodium (35 mg/kg). Deep anesthesia was maintained with additional intravenous injections of pentobarbital sodium (3.64 mg/kg/h). Rectal temperature was maintained at 37.6 °C with a thermostatically controlled heating pad. To ensure appropriate depth of anesthesia, electrocardiograms (ECG) were monitored throughout the procedure. On the first day of recording, during the surgery to expose the cortical surface in the chamber, the monkey was ventilated with 1.0–2.0% isoflurane in a mixture of 70% N_2_O and 30% O_2_ to maintain general anesthesia. ECG, electroencephalogram (EEG), end-tidal CO_2_ concentration, and rectal temperature were monitored throughout the experiment, and deep anesthesia was maintained by adjusting the concentration of isoflurane between 1.0 and 2.0%. The end-tidal CO_2_ concentration was maintained between 3.5 and 4.5%. During ISOI and electrophysiological recording, monkeys were paralyzed with intravenous vecuronium bromide (0.067 mg/kg/h) and ventilated with up to 0.5% isoflurane in a mixture of 70% N_2_O and 30% O_2_. For analgesia, fentanyl citrate (0.83 µg/kg/h) was infused intravenously and continued throughout the experiment.

After completion of the experiments and discontinuation of anesthesia and muscle relaxants, artificial ventilation was maintained until spontaneous breathing was observed. Once spontaneous breathing was confirmed, the animal was disconnected from the ventilator and maintained in a small recovery cage with a heating pad under close observation. After the animal showed signs of movement, it was returned to its home cage. The animal was continuously monitored until it recovered from anesthesia without complications and demonstrated normal behavioral patterns, including stable locomotion and regular feeding/drinking behavior.

### Surgical procedures

The first surgery involved the implantation of a titanium head post and recording chamber. The head post was attached to the top of the skull to immobilize the head during the experiments. Once attached, two stainless steel bolts for EEG recording were implanted through the skull above the dural surface of the left and right frontal cortices. An inverted T-shaped titanium bolt (T-bolt) was implanted through the skull for electrical grounding purposes at a distance from the EEG recording bolts; the flat side of the T-bolt was attached to the dural surface for stable grounding. Finally, a titanium chamber (18.0 mm inner diameter) was secured to the skull with dental cement so that the center of the chamber was positioned 15.0–20.0 mm anterior to the ear bar (Fig. 1A). In the next surgery, the skull and dura inside the chamber were largely removed. Subsequently, ISOI and extracellular recordings were performed over multiple days with an interval of 4–7 days. For ISOI, the chamber was filled with heavy silicone oil (1000 centistokes), and a glass coverslip was attached to the chamber as an imaging window (Fig. 1B). For extracellular recordings, the exposed cortex was covered with a clear artificial dura mater made of silicone sheet^70^. The chamber was then filled with 2.5% agarose containing dexamethasone (8.25 µg/ml) and antibiotics (25 µg/ml), and covered with a plastic coverslip with a small hole in it. The electrodes were inserted through this hole. The surface vascular pattern was used as a standard for mapping the site of electrode penetration.

The exposed cortex in the chamber corresponds mainly to the anterior and central inferior temporal cortex (AIT and CIT)^71^ and to the dorsal part of cytoarchitectonic area TE.

### Visual stimulation

Visual stimuli were presented monocularly to the eye contralateral to the recording hemisphere. The corneal curvature and optical power of the eye were examined, and based on these, appropriate contact lenses were used to focus the image presented at 57 cm from the cornea on the retina. Fundus photographs were taken to determine the location of the fovea. Several retinal landmarks, such as the intersection of blood vessels and the center of the optic disc, were projected onto the monitor using a retinoscope, and the position corresponding to the fovea on the monitor was determined geometrically by reference to the fundus photographs. The stimuli used were created by photographing various three-dimensional objects and cropping out the object parts from those images. To avoid stimulus bias, stimuli were selected from a variety of categories, including fruits, vegetables, plants, tools, animals, and stuffed animals, encompassing a variety of shapes and colors. Some of these were also used in our previous studies^39,72^. These visual stimuli were presented on a gray background on a 21-inch CRT monitor. The size of the stimuli was approximately 8–10 degrees of visual angle (dva). The average luminance was not measured. Depending on the monkey, geometrically simpler visual stimuli were also used. These stimuli were created in previous studies by extracting specific visual features from complex object images or using basic geometric shapes and simple patterns, designed to sufficiently activate neurons in area TE^42^. During stimulus presentation, the stimulus was moved along a circular path (with a radius of 0.4 dva, 1 cycle/s) centered on a location corresponding to the fovea. For ISOI, each trial began with a blank pre-stimulus period (1 s), followed by the presentation of a visual stimulus (2 s), and then a blank post-stimulus period (1 s). This was followed by an intertrial interval (10 s). In the electrophysiological recordings, the pre-stimulus period, the stimulus presentation period, the post-stimulus period, and the intertrial interval were 0.5–1 s, 1 s, 0.5–1 s, and 1 s, respectively. For both ISOI and electrophysiological recordings, 18–24 images were used in a single session and presented in a pseudorandom order. For ISOI, a gray blank screen was also presented as a control (blank condition). Each visual stimulus or blank was presented 32 times in the ISOI and 12 times in the electrophysiological recordings.

### ISOI recordings

The cortex inside the chamber was illuminated through a glass coverslip window with light at a wavelength of 605 nm. Reflected light from the cortex was captured by a CCD camera (XC-7500; SONY, Tokyo, Japan) with a tandem lens system^73^ mounted on a custom-made manipulator (Narishige, Tokyo, Japan), through a custom-designed neutral density filter that ensured uniform brightness across the cortex^74^. The camera focus was set at a depth of 500 μm from the cortical surface. Recordings were taken continuously for 4.0 s, starting 1.0 s before stimulus onset for each trial. The signal from the camera was digitized at 2 frames per second by a 10-bit video capture board (Pulsar, Matrox, Canada) and stored on a computer. The imaging area was 6.4 × 4.8 mm with a resolution of 320 × 240 pixels. Images of the cortical surface, including blood vessels, were obtained under 540-nm light illumination prior to the imaging session with stimuli.

### ISOI data analysis

The ISOI data analysis method has been described previously^75^. Briefly, the acquired image frames were analyzed offline using custom software written in MATLAB (MathWorks). For each stimulus presentation, a reflectance change map (Δ*R/R* map) was generated by averaging the frames for 1–2 s after stimulus onset, subtracting the average of the frames for 0–1 s before onset, and then dividing by this pre-stimulus average on a pixel-by-pixel basis. Then, to extract locally induced reflectance changes (mapping signal: ~0.5 mm) from stimulus-unspecific large-scale changes (global signal: > several mm)^75,76^, each Δ*R/R* map was convolved with a 1.6 × 1.6-mm median filter and subtracted from the original map (high-pass filtering). Finally, the Δ*R/R* maps were averaged across trials of a given stimulus condition, and from this average, the average of the Δ*R/R* maps obtained in the blank condition was subtracted (blank subtraction) to create a single condition map for that stimulus condition. Because an increase in neural activity induced by the stimulus leads to an increase in light absorption, which in turn results in a decrease in reflectance^76^, active sites appear darker in the single-condition maps.

A two-tailed Wilcoxon rank-sum test was used to identify regions that showed a significantly greater decrease in reflectance than the blank condition under visual stimulus conditions as stimulus-responsive regions. The *P* value was calculated from the statistics at each pixel and mapped (response map). To control for increasing type I errors due to multiple comparisons, only regions consisting of at least 100 contiguous pixels with *P* < 0.05 and containing pixels with *P* < 0.001 were considered to have significant signal changes, and regions that did not meet these criteria were excluded. To remove high spatial frequency noise from the response maps, each Δ*R/R* map was smoothed with a 100 × 100 μm median filter before statistical analysis. Signals from pixels on and near large vessels were less reliable because of large trial-by-trial variations that occurred even in the absence of visual stimulation. To exclude these regions from the analysis, we calculated the pixel-wise standard deviation (SD) of the blank condition images across trials. Pixels with large SDs (exceeding the upper bound of the 95% one-sided confidence interval based on the χ^2^ distribution) were excluded from further analysis (high SD regions, shaded dark green in the statistical maps). When calculating the optical response in the area of individual terminal patches, only patches composed of at least 100 consecutive pixels excluding high SD regions were analyzed, and the mean Δ*R/R* of the regions excluding high SD regions was calculated for each patch.

### Electrophysiological recordings

Tungsten microelectrodes (FHC, Bowdoin, Maine, catalog number UEWLEJTMNN1E) were used for electrophysiological recordings, either individually or in bundles. To create the bundles, the shafts of three electrodes, each 150 μm in diameter, were glued together so that the centers of the electrodes were approximately 150 μm apart from each other^39^. Based on the image of the surface vessels obtained during imaging, the electrode or bundle of electrodes was inserted perpendicularly into the cortical surface through an artificial silicone dura using an electrical microdrive (Narishige, Japan). Before insertion, the tip of the electrode was dipped several times into a solution of fluorescent dye DiI (Molecular Probes; 10% in dehydrated ethanol). This marked the cortical surface at the insertion site with DiI, facilitating identification of the recording position in the pattern of surface blood vessels. The electrode was advanced until the first spiking activity was observed. The depth at which this initial spiking activity was found was set as the baseline depth (0 μm). Neuronal activity was extracellularly recorded every 250 μm as the electrode was advanced. Recordings were made at each depth after a 20-minute waiting period to ensure that the recording position was stable. Recordings were made for a period of 2–3 s starting 0.5–1 s before stimulus onset for each trial. At each electrode insertion site, recordings were made at three depths ranging from 0 to 500 μm. The raw electrical signals from the electrode were amplified and passed through a band-pass filter (filter range, 500 Hz to 10 kHz). The filtered signals were digitized at 25,000 Hz and stored on a computer.

### MUA analysis

Electrical signals that exceeded 3.5 times the standard deviation (SD) of the baseline were considered multi-unit activity (MUA) recorded extracellularly by the electrode from multiple cells. The trial-by-trial average of MUA recorded at multiple depths from 0 to 500 μm from the reference depth was calculated, and this was regarded as the average MUA in the supragranular layers of the recorded site (averaged MUA). The evoked response of the averaged MUA to each stimulus was calculated by subtracting the average firing rate during the 0.5- or 1-s period before stimulus onset from the average firing rate during the 1-s period starting 80 ms after stimulus onset. The evoked response was averaged over 12 trials. The significance of the evoked responses was tested using a two-sided Wilcoxon signed-rank test (significance level, *P* = 0.05, *n* = 12 trials).

### Tracer injections and marking with DiI

The injection procedure followed the methods described in previous studies^6,48^. Briefly, in anesthetized monkeys, the chamber cap was opened to access the cortex. Then, based on images of surface blood vessels, a glass micropipette was inserted at the target site, with the tip positioned at a depth of 0.6–0.7 mm, corresponding to layer 3 of the cortex. Each micropipette was filled with a solution of either biotinylated dextran amine (BDA, a mixture of 10,000 and 3,000 MW in equal volumes, 10% in 0.1 M phosphate buffer, pH 7.4; Molecular Probes) or tetramethylrhodamine-conjugated dextran amine (Fluoro-Ruby, FR, a mixture of 10,000 and 3,000 MW in equal volumes, 10% in 0.1 M phosphate buffer, pH 7.4; Molecular Probes), both of which are primarily used as anterograde tracers. The pipette tip had an inner diameter of 20–25 μm. We administered these tracers iontophoretically, using a positive current of 3–7 µA in a cycle of 7 s on and 7 s off, over a period of 5–10 min.

Prior to perfusion, a metal needle dipped several times in DiI solution was inserted at multiple locations (at least four) within the area where ISOI had been performed. First, the chamber cap was opened to expose the cortex, and the CCD camera used for ISOI, attached to a manipulator, was positioned to focus on the cortical surface of the imaged area. A custom-designed needle holder was then attached to the CCD camera. This holder was designed to be parallel to the optical axis of the lens, allowing the needle to be inserted perpendicular to the imaged plane, the cortical surface, by controlling the manipulator. The needle was slowly inserted into the cortex to a depth of 3 mm, left in place for 5 min, and then withdrawn.

### Histology

After a survival period of 7–13 days following tracer injection, the monkey was deeply anesthetized with an overdose of sodium pentobarbital (75 mg/kg, i.p.) after induction anesthesia with ketamine (5.8 mg/kg, i.m.). The animal was perfused transcardially with 1 L of 0.1 M phosphate-buffered saline (PBS, pH 7.4, 37 °C), followed by 4 L of 4% paraformaldehyde in 0.1 M phosphate buffer (PB; pH 7.4, 4 °C), and finally with 1 L each of 10%, 20%, and 30% sucrose in 0.1 M PB (4 °C). The brain was then removed, and a block of cortical tissue containing the recording and injection sites was excised. The tissue block was then immersed in 30% sucrose in PB (4 °C) for two days. The block was flattened by applying pressure to the cortical surface with a glass slide during the freezing process using dry ice. It was then sectioned tangentially to the cortical surface at a thickness of 50 μm using a freezing microtome. This ensured that the cut surfaces were parallel to the surface. The cut sections were collected in 0.1 M PBS (4 °C).

For BDA staining, a series of sections was incubated overnight on a shaker at 4 °C in avidin-biotin complex (ABC) solution (Vectastain, Elite ABC kit; Vector) with 0.4% Triton X-100 (Sigma, St. Louis, MO). After washing with 0.1 M PBS, sections were incubated at room temperature in 0.1 M PBS containing 0.035% diaminobenzidine hydrochloride (DAB; Sigma), 0.03% ammonium nickel sulfate, and 0.0004% hydrogen peroxide. The reaction was terminated by thorough washing in PBS after the appearance of the black BDA reaction product. Sections were mounted on gelatin-coated slides, dried, dehydrated in ethanol, cleared in Hemo-De (Fischer Scientific, Chicago, IL), and coverslipped with Entellan (Merck, Darmstadt, Germany). Another series of sections was used for Nissl staining to determine layer boundaries.

### Tracer labeling analysis

Sections were imaged under bright-field illumination with a digital camera (AxioCam HRc, Zeiss, Germany) attached to a microscope (AxioScope 2 plus, Zeiss, Germany). Individual images taken with a 10× objective lens were imported into Photoshop software (Adobe, San Jose, CA), processed, and assembled into larger montage images that included the injection site and labeled horizontal axonal terminal patches. Image processing included enlargement, reduction, rotation, and brightness and contrast adjustments, which were applied uniformly to the images.

Axon terminal patches were identified as clusters of labeled axon terminals observed across consecutive sections. The section through the supragranular layer with the most well-stained terminal patches was selected, and terminal patch extraction was performed from its montage image using Photoshop. First, retrogradely labeled cells and blood vessels were filtered out of the image. Then, an edge detection algorithm, which detects the maximum local brightness change on the filtered image using the difference of two Gaussian filters (radii 75 μm and 120 μm), was applied to delineate the terminal patches (for details, see Ref. 6).

It was difficult to estimate the extent of the injection site (effective tracer uptake region) due to the large amount of labeling around the injection site. Therefore, we estimated the extent of the injection site from the spread of the distribution of descending axon bundles labeled in deeper sections, because these axon bundles are thought to originate from neurons labeled in the tracer uptake region in the upper layers^43^.

### Alignment of functional maps and terminal patches

To align the positions of terminal patches and electrophysiological recording sites with functional maps obtained by intrinsic signal optical imaging (ISOI), the following steps were taken. Prior to perfusion, metal needles coated with DiI solution were vertically inserted into multiple sites on the cortical surface as described above. The insertion sites and surrounding blood vessel patterns were photographed through a surgical microscope with a digital camera. These images were used to identify the insertion sites on the functional maps (Fig. 2F). In the tangential sections prepared after perfusion, the insertion sites of the DiI-coated needles were easily identifiable as DiI-labeled spots or small lesions (Fig. 2E). To correct for tissue distortion and shrinkage that would have occurred during section preparation, we performed a local alignment procedure. For each terminal patch identified using the edge detection algorithm, we selected three nearby reference points consisting of either DiI insertion sites or the center of the injection site. Using these reference points, we performed image processing that included only linear transformations to align each terminal patch in the section image with its corresponding location on the functional map obtained in vivo. This local alignment procedure allowed us to minimize the effects of non-linear distortion and to compensate for the effects of tissue shrinkage. The actual alignment was performed using Adobe Photoshop software.

To test whether the number of patches activated by at least one of the object stimuli in the same way as the injection site and the proportion of patch regions within the imaged area that overlapped with regions where responses correlated with the injection site (overlap ratio) were significantly greater than chance level, we performed a one-tailed randomization test. In this test, the locations of all patches were randomly shifted within the imaged area, ensuring no overlap between patches. The overlap ratio between these randomly shifted patches and the response correlation regions of the injection site was then calculated. This process was repeated 10,000 times to obtain a null distribution of overlap ratios. The *P* value was calculated as the proportion of iterations in the null distribution that yielded an overlap ratio greater than the observed overlap ratio.

### Statistical analysis

All statistical analyses were performed using custom software written in MATLAB (Mathworks). The statistical significance level was set at *P* < 0.05. For detailed statistical methods and multiple comparison corrections, please refer to the relevant sections of the Methods.

## Electronic supplementary material

Below is the link to the electronic supplementary material.

Supplementary Material 1

## Data Availability

All data in this study are available from the corresponding author upon reasonable request.

## References

[1] Gilbert, C. D. & Wiesel, T. N. Morphology and intracortical projections of functionally characterised neurones in the cat visual cortex. *Nature* **280**, 120–125 (1979). 552600 10.1038/280120a0

[2] Rockland, K. S. & Lund, J. S. Widespread periodic intrinsic connections in the tree shrew visual cortex. *Science* **215**, 1532–1534 (1982). 7063863 10.1126/science.7063863

[3] Amir, Y., Harel, M. & Malach, R. Cortical hierarchy reflected in the organization of intrinsic connections in macaque monkey visual cortex. *J. Comp. Neurol.* **334**, 19–46 (1993). 8408757 10.1002/cne.903340103

[4] Lund, J. S., Yoshioka, T. & Levitt, J. B. Comparison of intrinsic connectivity in different areas of macaque monkey cerebral cortex. *Cereb. Cortex* **3**, 148–162 (1993). 8490320 10.1093/cercor/3.2.148

[5] Kritzer, M. F. & Goldman-Rakic, P. S. Intrinsic circuit organization of the major layers and sublayers of the dorsolateral prefrontal cortex in the rhesus monkey. *J. Comp. Neurol.* **359**, 131–143 (1995). 8557842 10.1002/cne.903590109

[6] Tanigawa, H., Wang, Q. & Fujita, I. Organization of horizontal axons in the inferior temporal cortex and primary visual cortex of the macaque monkey. *Cereb. Cortex* **15**, 1887–1899 (2005). 15758199 10.1093/cercor/bhi067

[7] Malach, R., Amir, Y., Harel, M. & Grinvald, A. Relationship between intrinsic connections and functional architecture revealed by optical imaging and in vivo targeted biocytin injections in primate striate cortex. *Proc. Natl. Acad. Sci.* **90**, 10469–10473 (1993). 8248133 10.1073/pnas.90.22.10469 PMC47798

[8] Stettler, D. D., Das, A., Bennett, J. & Gilbert, C. D. Lateral connectivity and contextual interactions in macaque primary visual cortex. *Neuron* **36**, 739–750 (2002). 12441061 10.1016/s0896-6273(02)01029-2

[9] Rockland, K. S. & Lund, J. S. Intrinsic laminar lattice connections in primate visual cortex. *J. Comp. Neurol.* **216**, 303–318 (1983). 6306066 10.1002/cne.902160307

[10] Hubel, D. H. & Wiesel, T. N. Sequence regularity and geometry of orientation columns in the monkey striate cortex. *J. Comp. Neurol.* **158**, 267–293 (1974). 4436456 10.1002/cne.901580304

[11] Gilbert, C. D. & Wiesel, T. Columnar specificity of intrinsic horizontal and corticocortical connections in cat visual cortex. *J. Neurosci.* **9**, 2432–2442 (1989). 2746337 10.1523/JNEUROSCI.09-07-02432.1989 PMC6569760

[12] Bosking, W. H., Zhang, Y., Schofield, B. & Fitzpatrick, D. Orientation selectivity and the arrangement of horizontal connections in tree shrew striate cortex. *J. Neurosci.* **17**, 2112–2127 (1997). 9045738 10.1523/JNEUROSCI.17-06-02112.1997 PMC6793759

[13] Tootell, R., Silverman, M., Hamilton, S., Valois, R. D. & Switkes, E. Functional anatomy of macaque striate cortex. III. Color. *J. Neurosci.* **8**, 1569–1593 (1988). 3367211 10.1523/JNEUROSCI.08-05-01569.1988 PMC6569202

[14] Tootell, R., Silverman, M., Hamilton, S., Switkes, E. & DeValois, R. Functional anatomy of macaque striate cortex. V. spatial frequency. *J. Neurosci.* **8**, 1610–1624 (1988). 3367213 10.1523/JNEUROSCI.08-05-01610.1988 PMC6569194

[15] Livingstone, M. S. & Hubel, D. H. Anatomy and physiology of a color system in the primate visual cortex. *J. Neurosci.* **4**, 309–356 (1984). 6198495 10.1523/JNEUROSCI.04-01-00309.1984 PMC6564760

[16] Livingstone, M. & Hubel, D. Specificity of intrinsic connections in primate primary visual cortex. *J. Neurosci.* **4**, 2830–2835 (1984). 6209365 10.1523/JNEUROSCI.04-11-02830.1984 PMC6564722

[17] Ts’o, D. Y., Frostig, R. D., Lieke, E. E. & Grinvald, A. Functional organization of primate visual cortex revealed by high resolution optical imaging. *Science* **249**, 417–420 (1990). 2165630 10.1126/science.2165630

[18] Yoshioka, T., Blasdel, G. G., Levitt, J. B. & Lund, J. S. Relation between patterns of intrinsic lateral connectivity, ocular dominance, and cytochrome oxidase-reactive regions in macaque monkey striate cortex. *Cereb. Cortex* **6**, 297–310 (1996). 8670658 10.1093/cercor/6.2.297

[19] Hebb, D. O. *The Organization of Behavior; a Neuropsychological Theory* (Wiely, 1949).

[20] Löwel, S. & Singer, W. Selection of intrinsic horizontal connections in the visual cortex by correlated neuronal activity. *Science* **255**, 209–212 (1992). 1372754 10.1126/science.1372754

[21] Schmidt, K. E., Kim, D. S., Singer, W., Bonhoeffer, T. & Löwel, S. Functional specificity of long-range intrinsic and interhemispheric connections in the visual cortex of strabismic cats. *J. Neurosci.* **17**, 5480–5492 (1997). 9204930 10.1523/JNEUROSCI.17-14-05480.1997 PMC6793806

[22] Gilbert, C. D. & Li, W. Top-down influences on visual processing. *Nat. Rev. Neurosci.* **14**, 350–363 (2013). 23595013 10.1038/nrn3476 PMC3864796

[23] Kisvárday, Z. F., Tóth, E., Rausch, M. & Eysel, U. T. Orientation-specific relationship between populations of excitatory and inhibitory lateral connections in the visual cortex of the cat. *Cereb. Cortex* **7**, 605–618 (1997). 9373017 10.1093/cercor/7.7.605

[24] Buzás, P. et al. Model-based analysis of excitatory lateral connections in the visual cortex. *J. Comp. Neurol.* **499**, 861–881 (2006). 17072837 10.1002/cne.21134

[25] Martin, K. A. C., Roth, S. & Rusch, E. S. Superficial layer pyramidal cells communicate heterogeneously between multiple functional domains of cat primary visual cortex. *Nat. Commun.* **5**, 5252 (2014). 25341917 10.1038/ncomms6252 PMC4354012

[26] Chavane, F. et al. Lateral spread of orientation selectivity in V1 is controlled by intracortical cooperativity. *Front. Syst. Neurosci.* **5**, 4 (2011). 21629708 10.3389/fnsys.2011.00004 PMC3100672

[27] Huang, X., Elyada, Y. M., Bosking, W. H., Walker, T. & Fitzpatrick, D. Optogenetic assessment of horizontal interactions in primary visual cortex. *J. Neurosci.* **34**, 4976–4990 (2014). 24695715 10.1523/JNEUROSCI.4116-13.2014 PMC3972723

[28] Levitt, J. B., Yoshioka, T. & Lund, J. S. Intrinsic cortical connections in macaque visual area V2: evidence for interaction between different functional streams. *J. Comp. Neurol.* **342**, 551–570 (1994). 8040365 10.1002/cne.903420405

[29] Malach, R., Tootell, R. B. H. & Malonek, D. Relationship between orientation domains, cytochrome oxidase stripes, and intrinsic horizontal connections in squirrel monkey area V2. *Cereb. Cortex* **4**, 151–165 (1994). 8038566 10.1093/cercor/4.2.151

[30] Malach, R., Schirman, T. D., Harel, M., Tootell, R. B. & Malonek, D. Organization of intrinsic connections in owl monkey area MT. *Cereb. Cortex* **7**, 386–393 (1997). 9177768 10.1093/cercor/7.4.386

[31] Chavane, F., Perrinet, L. U. & Rankin, J. Revisiting horizontal connectivity rules in V1: from like-to-like towards like-to-all. *Brain Struct. Funct.* **227**, 1279–1295 (2022). 35122520 10.1007/s00429-022-02455-4

[32] Rockland, K. S. Clustered intrinsic connections: not a single system. *Front. Syst. Neurosci.* **16**, 910845 (2022). 35720440 10.3389/fnsys.2022.910845 PMC9203679

[33] Kisvárday, Z. Academic Press,. Topography of excitatory cortico-cortical connections in three main tiers of the visual cortex: functional implications of the patchy horizontal network. in *Axons and Brain Architecture* (ed. Rockland, K. S.) 135–158 (2016).

[34] von Bonin, G. & Bailey, P. *The Neocortex of Macaca Mulatta* (1947).

[35] Kravitz, D. J., Saleem, K. S., Baker, C. I., Ungerleider, L. G. & Mishkin, M. The ventral visual pathway: an expanded neural framework for the processing of object quality. *Trends Cogn. Sci.* **17**, 26–49 (2013). 23265839 10.1016/j.tics.2012.10.011 PMC3532569

[36] Gross, C. G., Rocha-Miranda, C. E. & Bender, D. B. Visual properties of neurons in inferotemporal cortex of the Macaque. *J. Neurophysiol.* **35**, 96–111 (1972). 4621506 10.1152/jn.1972.35.1.96

[37] Desimone, R., Albright, T. D., Gross, C. G. & Bruce, C. Stimulus-selective properties of inferior temporal neurons in the macaque. *J. Neurosci.* **4**, 2051–2062 (1984). 6470767 10.1523/JNEUROSCI.04-08-02051.1984 PMC6564959

[38] Tanaka, K., Saito, H., Fukada, Y. & Moriya, M. Coding visual images of objects in the inferotemporal cortex of the macaque monkey. *J. Neurophysiol.* **66**, 170–189 (1991). 1919665 10.1152/jn.1991.66.1.170

[39] Sato, T., Uchida, G. & Tanifuji, M. Cortical columnar organization is reconsidered in inferior temporal cortex. *Cereb. Cortex* **19**, 1870–1888 (2009). 19068487 10.1093/cercor/bhn218 PMC2705700

[40] Fujita, I., Tanaka, K., Ito, M. & Cheng, K. Columns for visual features of objects in monkey inferotemporal cortex. *Nature* **360**, 343–346 (1992). 1448150 10.1038/360343a0

[41] Tsunoda, K., Yamane, Y., Nishizaki, M. & Tanifuji, M. Complex objects are represented in macaque inferotemporal cortex by the combination of feature columns. *Nat. Neurosci.* **4**, 832–838 (2001). 11477430 10.1038/90547

[42] Wang, G., Tanaka, K. & Tanifuji, M. Optical imaging of functional organization in the monkey inferotemporal cortex. *Science* **272**, 1665–1668 (1996). 8658144 10.1126/science.272.5268.1665

[43] Fujita, I. & Fujita, T. Intrinsic connections in the macaque inferior temporal cortex. *J. Comp. Neurol.* **368**, 467–486 (1996). 8744437 10.1002/(SICI)1096-9861(19960513)368:4<467::AID-CNE1>3.0.CO;2-2

[44] Tanaka, K. Inferotemporal cortex and object vision. *Annu. Rev. Neurosci.* **19**, 109–139 (1996). 8833438 10.1146/annurev.ne.19.030196.000545

[45] Tsao, D. Y., Freiwald, W. A., Knutsen, T. A., Mandeville, J. B. & Tootell, R. B. H. Faces and objects in macaque cerebral cortex. *Nat. Neurosci.* **6**, 989–995 (2003). 12925854 10.1038/nn1111 PMC8117179

[46] Tanigawa, H., Fujita, I., Kato, M. & Ojima, H. Distribution, morphology, and γ-aminobutyric acid immunoreactivity of horizontally projecting neurons in the macaque inferior temporal cortex. *J. Comp. Neurol.* **401**, 129–143 (1998). 9802704 10.1002/(sici)1096-9861(19981109)401:1<129::aid-cne8>3.0.co;2-d

[47] Tamura, H., Kaneko, H. & Fujita, I. Quantitative analysis of functional clustering of neurons in the macaque inferior temporal cortex. *Neurosci. Res.* **52**, 311–322 (2005). 15893835 10.1016/j.neures.2005.04.006

[48] Wang, Q., Tanigawa, H. & Fujita, I. Postnatal development of intrinsic horizontal axons in macaque inferior temporal and primary visual cortices. *Cereb. Cortex* **27**, 2708–2726 (2017). 27114175 10.1093/cercor/bhw105

[49] Coogan, T. A. & Essen, D. C. V. Development of connections within and between areas V1 and V2 of macaque monkeys. *J. Comp. Neurol.* **372**, 327–342 (1996). 8873864 10.1002/(SICI)1096-9861(19960826)372:3<327::AID-CNE1>3.0.CO;2-4

[50] Ackman, J. B., Burbridge, T. J. & Crair, M. C. Retinal waves coordinate patterned activity throughout the developing visual system. *Nature* **490**, 219–225 (2014). 10.1038/nature11529 PMC3962269 23060192

[51] Kim, J., Song, M., Jang, J. & Paik, S. B. Spontaneous retinal waves can generate long-range horizontal connectivity in visual cortex. *J. Neurosci.* **40**, 6584–6599 (2020). 32680939 10.1523/JNEUROSCI.0649-20.2020 PMC7486661

[52] Arcaro, M. J. & Livingstone, M. S. A hierarchical, retinotopic proto-organization of the primate visual system at birth. *Elife* **6**, e26196 (2017). 28671063 10.7554/eLife.26196 PMC5495573

[53] Arcaro, M. J. & Livingstone, M. S. On the relationship between maps and domains in inferotemporal cortex. *Nat. Rev. Neurosci.* **22**, 573–583 (2021). 34345018 10.1038/s41583-021-00490-4 PMC8865285

[54] Livingstone, M. S. et al. Development of the macaque face-patch system. *Nat. Commun.* **8**, 14897 (2017). 28361890 10.1038/ncomms14897 PMC5381009

[55] Arcaro, M. J., Schade, P. F., Vincent, J. L., Ponce, C. R. & Livingstone, M. S. Seeing faces is necessary for face-domain formation. *Nat. Neurosci.* **20**, 1404–1412 (2017). 28869581 10.1038/nn.4635 PMC5679243

[56] Srihasam, K., Vincent, J. L. & Livingstone, M. S. Novel domain formation reveals proto-architecture in inferotemporal cortex. *Nat. Neurosci.* **17**, 1776–1783 (2014). 25362472 10.1038/nn.3855 PMC4241119

[57] Priebe, N. J. Mechanisms of orientation selectivity in the primary visual cortex. *Annu. Rev. Vis. Sci.* **2**, 85–107 (2016). 28532362 10.1146/annurev-vision-111815-114456

[58] Kaas, J. H. & Lyon, D. C. Pulvinar contributions to the dorsal and ventral streams of visual processing in primates. *Brain Res. Rev.* **55**, 285–296 (2007). 17433837 10.1016/j.brainresrev.2007.02.008 PMC2100380

[59] Stettler, D. D., Yamahachi, H., Li, W., Denk, W. & Gilbert, C. D. Axons and synaptic boutons are highly dynamic in adult visual cortex. *Neuron* **49**, 877–887 (2006). 16543135 10.1016/j.neuron.2006.02.018

[60] Li, W., Piëch, V. & Gilbert, C. D. Contour saliency in primary visual cortex. *Neuron* **50**, 951–962 (2006). 16772175 10.1016/j.neuron.2006.04.035

[61] McManus, J. N. J., Li, W. & Gilbert, C. D. Adaptive shape processing in primary visual cortex. *Proc. Natl. Acad. Sci.* **108**, 9739–9746 (2011). 21571645 10.1073/pnas.1105855108 PMC3116391

[62] Naya, Y., Yoshida, M. & Miyashita, Y. Backward spreading of memory-retrieval signal in the primate temporal cortex. *Science* **291**, 661–664 (2001). 11158679 10.1126/science.291.5504.661

[63] Chelazzi, L., Duncan, J., Miller, E. K. & Desimone, R. Responses of neurons in inferior temporal cortex during memory-guided visual search. *J. Neurophysiol.* **80**, 2918–2940 (1998). 9862896 10.1152/jn.1998.80.6.2918

[64] Freedman, D. J., Riesenhuber, M., Poggio, T. & Miller, E. K. A comparison of primate prefrontal and inferior temporal cortices during visual categorization. *J. Neurosci.* **23**, 5235–5246 (2003). 12832548 10.1523/JNEUROSCI.23-12-05235.2003 PMC6741148

[65] Koida, K. & Komatsu, H. Effects of task demands on the responses of color-selective neurons in the inferior temporal cortex. *Nat. Neurosci.* **10**, 108–116 (2006). 17173044 10.1038/nn1823

[66] DiCarlo, J. J., Zoccolan, D. & Rust, N. C. How does the brain solve visual object recognition? *Neuron* **73**, 415–434 (2012). 22325196 10.1016/j.neuron.2012.01.010 PMC3306444

[67] Wang, Y., Fujita, I. & Murayama, Y. Neuronal mechanisms of selectivity for object features revealed by blocking inhibition in inferotemporal cortex. *Nat. Neurosci.* **3**, 807–813 (2000). 10903574 10.1038/77712

[68] Hirsch, J. & Gilbert, C. Synaptic physiology of horizontal connections in the cat’s visual cortex. *J. Neurosci.* **11**, 1800–1809 (1991). 1675266 10.1523/JNEUROSCI.11-06-01800.1991 PMC6575415

[69] Verhoef, B. E., Vogels, R. & Janssen, P. Inferotemporal cortex subserves three-dimensional structure categorization. *Neuron* **73**, 171–182 (2012). 22243755 10.1016/j.neuron.2011.10.031

[70] Arieli, A., Grinvald, A. & Slovin, H. Dural substitute for long-term imaging of cortical activity in behaving monkeys and its clinical implications. *J. Neurosci. Methods* **114**, 119–133 (2002). 11856563 10.1016/s0165-0270(01)00507-6

[71] Felleman, D. J. & Essen, D. C. V. distributed hierarchical processing in the primate cerebral cortex. *Cereb. Cortex* **1**, 1–47 (1991). 1822724 10.1093/cercor/1.1.1-a

[72] Sato, T. et al. Object representation in inferior temporal cortex is organized hierarchically in a mosaic-like structure. *J. Neurosci.* **33**, 16642–16656 (2013). 24133267 10.1523/JNEUROSCI.5557-12.2013 PMC6618530

[73] Ratzlaff, E. H. & Grinvald, A. A tandem-lens epifluorescence macroscope: hundred-fold brightness advantage for wide-field imaging. *J. Neurosci. Methods* **36**, 127–137 (1991). 1905769 10.1016/0165-0270(91)90038-2

[74] Przybyszewski, A. W., Sato, T. & Fukuda, M. Optical filtering removes non-homogenous illumination artifacts in optical imaging. *J. Neurosci. Methods* **168**, 140–145 (2008). 10.1016/j.jneumeth.2007.09.006 17959253

[75] Tanigawa, H., Chen, G. & Roe, A. W. Spatial distribution of attentional modulation at columnar resolution in macaque area V4. *Front. Neural Circuits* **10**, 102 (2016). 28018181 10.3389/fncir.2016.00102 PMC5149540

[76] Malonek, D. & Grinvald, A. Interactions between electrical activity and cortical microcirculation revealed by imaging spectroscopy: implications for functional brain mapping. *Science* **272**, 551–554 (1996). 8614805 10.1126/science.272.5261.551

