# Supplementary material for: Relationship between functional structures and horizontal connections in macaque inferior temporal cortex
Source: Sci Rep. 2025 Jan 27;15:3436. doi: 10.1038/s41598-025-87517-3 (PMC11772672; doi:10.1038/s41598-025-87517-3)
Supplement: Supplementary file 1 — Supplementary Material 1 [file 41598_2025_87517_MOESM1_ESM.pdf]

Supplemental information

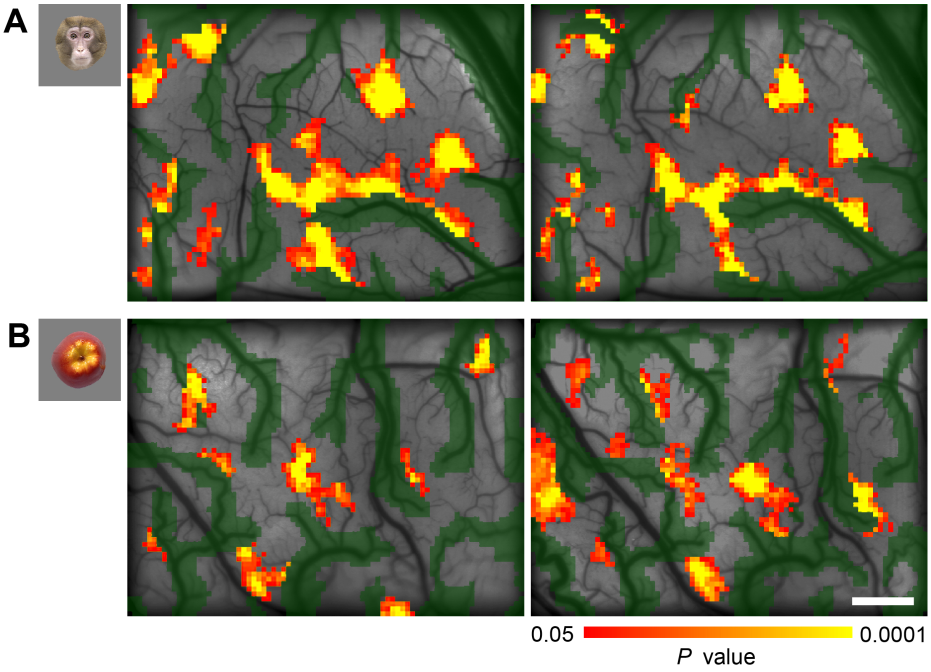

Figure S1. Stability of response maps to stimuli across days. (**A**) Response maps to a specific stimulus (monkey face) in the TE area of one monkey (M4) recorded on different days. The convention is the same as in Figure 1F. The left map is the same as in Figure 1F, and the right map is based on data from an imaging session 10 days later. (**B**) Response maps to a different stimulus (apple) in another monkey (M3). The right map was obtained in a session 4 days after the session in which the left map was obtained. The imaging ranges of both maps were slightly shifted, but the distribution of activation spots relative to the vascular pattern is largely similar. The scale bar is 1 mm for all panels in **A** and **B**.

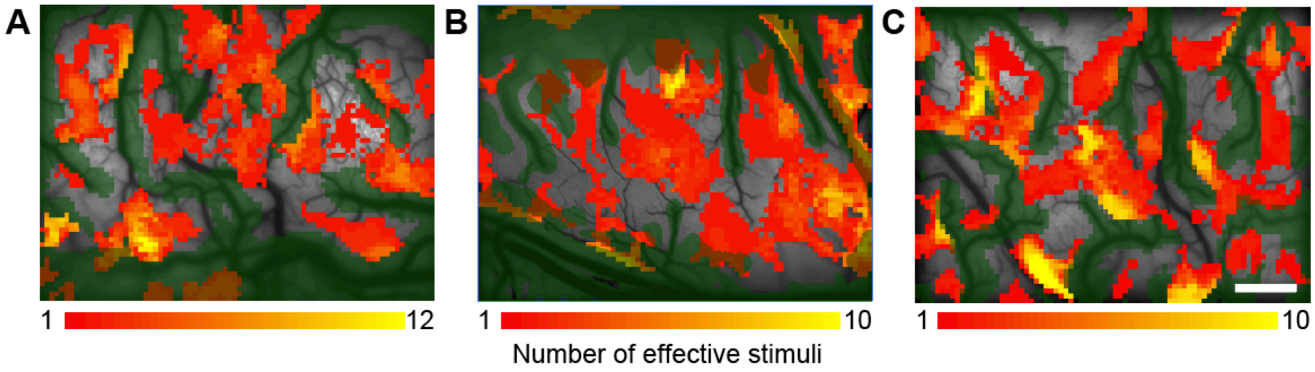

Figure S2. Maps showing the number of effective stimuli in monkeys other than M4. Maps indicating the number of stimuli from the used stimulus set that elicited significant responses in monkeys (**A**: M1, **B**: M2, **C**: M3) during imaging sessions. The convention is the same as in Figure 1K. The number of object stimuli used was 18 for M1, 20 for M2, and 24 for M3. The scale bar is 1mm.

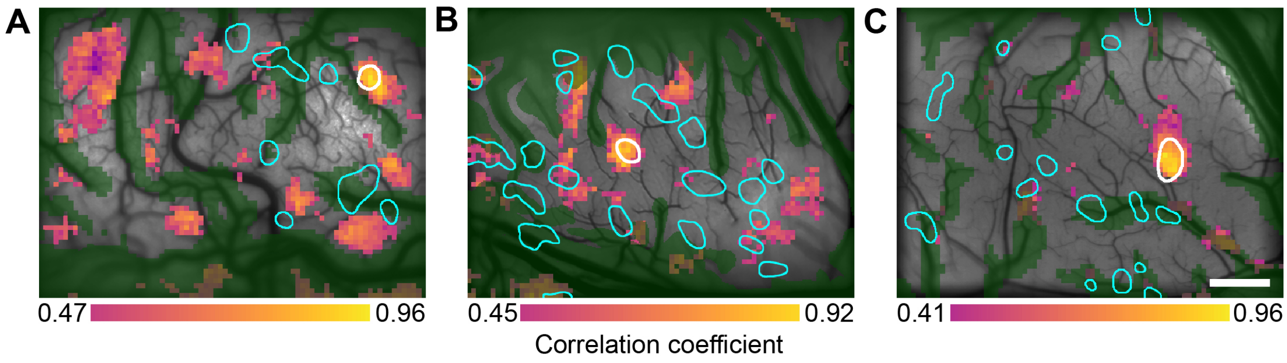

Figure S3. Response correlation maps in monkeys other than M3. Response correlation maps based on responses to the stimulus set used in the imaging sessions for monkeys (**A**: M1, **B**: M2, **C**: M4). Conventions are the same as in Figure 3C. Scale bar: 1 mm.

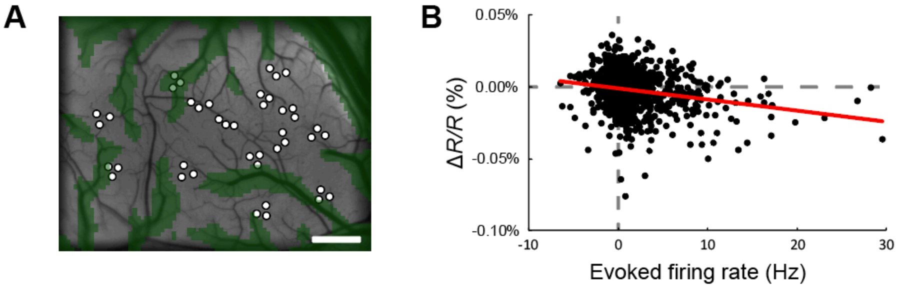

Figure S4. Correlation between optical imaging and electrophysiological responses in monkeys M4. (**A**) Electrode insertion sites within the imaging region in monkey M4. (**B**) Correlation between the mean response of the average MUA and the mean change in optical reflectance in imaging at the electrode insertion positions, in response to the object stimuli within the stimulus set. The regression line (red line) was $y = -8\times10^{-6}\,x - 1\times10^{-5}$. Other conventions are the same as in Figure 5B,C, respectively.
